# Supplementary material for: An Adrenalectomy Mouse Model Reflecting Clinical Features for Chronic Fatigue Syndrome
Source: Biomolecules. 2020 Jan 1;10(1):71. doi: 10.3390/biom10010071 (PMC7023174; doi:10.3390/biom10010071)
Supplement: Supplementary file 1 [file biomolecules-10-00071-s001.pdf]

Supplementary Material

Material and Methods

Sample preparation

All mice were sacrificed under CO_2 anesthesia through removal of blood from an abdominal vein. A partial blood sample was used for complete blood count analysis using hematological autoanalyzer (Exigo EOS Vet, Boule Medical AB, Sweden).

Results

Body weights

The body weights between sham and ADX groups were not significantly changed during 7 days (Supplementary Figure 2).

Hematological parameters

The blood cell population was changed by LPS and poly I:C injection. The LPS significantly reduced absolute WBC count [$F(3,20) = 7.840$, $p < 0.01$] compared with the vehicle ($p < 0.05$ or $p < 0.01$). Its components including lymphocyte [$F(3,20) = 22.859$, $p < 0.01$], monocyte [$F(3,20) = 8.266$, $p < 0.01$], and granulocyte [$F(3,20) = 20.796$, $p < 0.01$] were significantly altered by LPS and poly I:C injection ($p < 0.05$ or $p < 0.01$, Supplementary Table 1). Absolute and percentage of blood cells were not affected by ADX compared with both the vehicle and sham group.

Supplementary Table S1. Hematological parameters in the blood of mice.

Treatment	Vehicle	LPS	Poly I:C	Sham	ADX
RBC (10^{12}/L)	7.98 ± 0.27	7.79 ± 0.46	7.69 ± 0.49	7.36 ± 0.31	7.79 ± 0.44
Hemoglobin (g/dL)	14.42 ± 0.29	14.05 ± 0.79	14.01 ± 0.84	12.21 ± 0.35	14.12 ± 1.01
WBC (10^{9}/L)	14.28 ± 2.29	5.53 ± 1.73**	12.86 ± 2.44	13.51 ± 2.63	11.65 ± 5.69
Lymphocyte (%)	65.85 ± 2.64	31.45 ± 12.83**	43.50 ± 14.11*	66.26 ± 4.00	70.92 ± 6.19
Monocyte (%)	6.50 ± 0.28	10.60 ± 2.90*	8.76 ± 1.51*	7.38 ± 0.51	6.70 ± 0.71
Granulocyte (%)	27.65 ± 2.77	57.95 ± 12.22**	47.74 ± 12.99*	26.36 ± 3.55	22.38 ± 5.59

Data are expressed as the mean ± SD (n = 6) *P < 0.05, **P < 0.01 compared with the vehicle group. RBC, red blood cell; WBC, white blood cell

Supplementary Figures

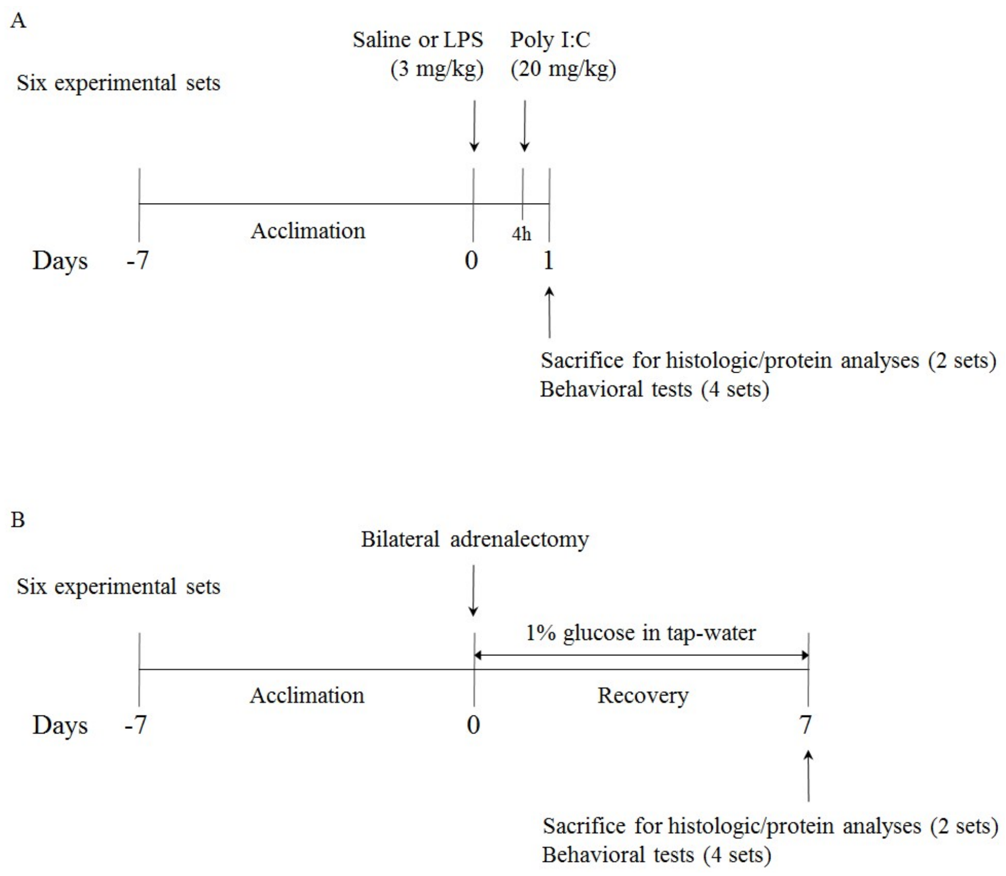

Supplementary Figure S1. Experimental schedule. ICR mouse (8 weeks old) was injected to LPS (3 mg/kg for 24 h) or poly I:C (20 mg/kg for 4 h) intraperitoneally (A). Other mice were subjected to bilateral adrenalectomy and then they were allowed to rest with 1% glucose for 7 days (B).

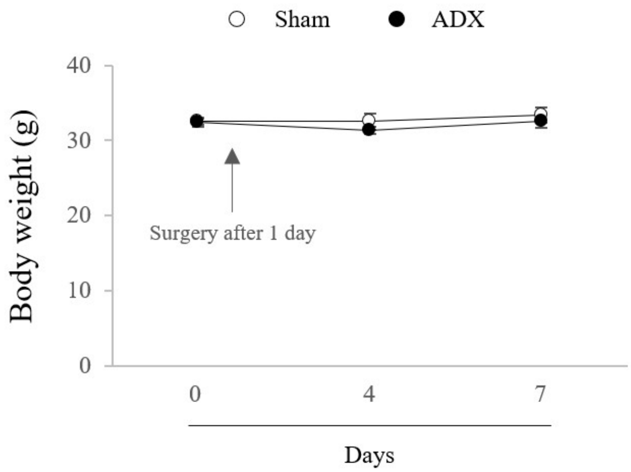

Supplementary Figure S2. Changes in body weight of the sham or ADX mice during recovery. The body weight of sham (open circles) and ADX (filled circles) was measured during 7 days.

Figure 5A full-length gel and blots

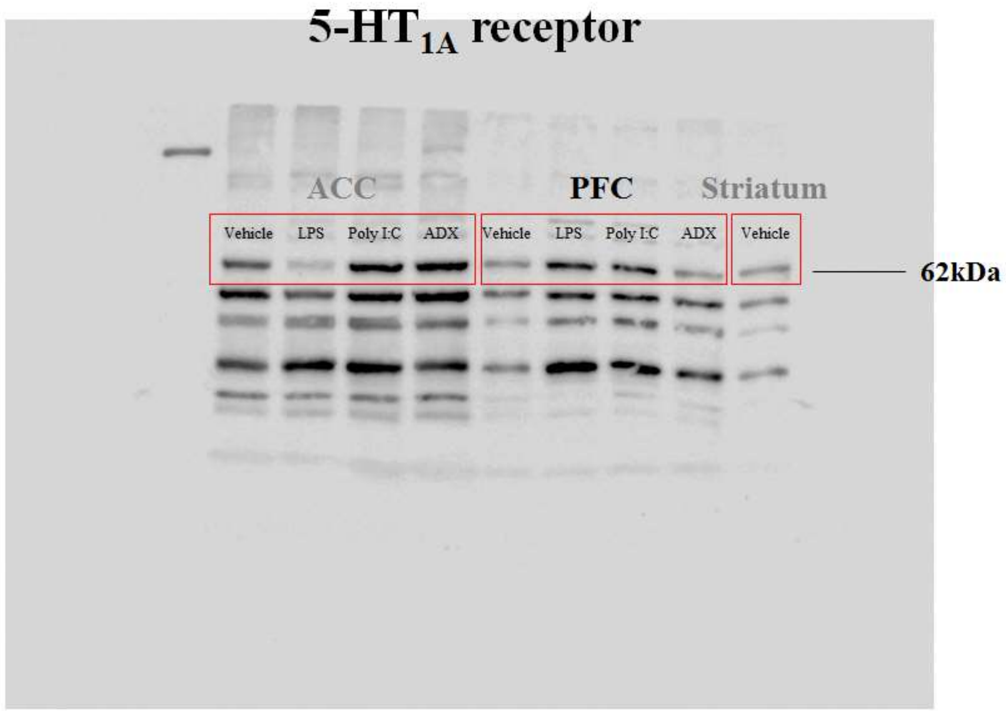

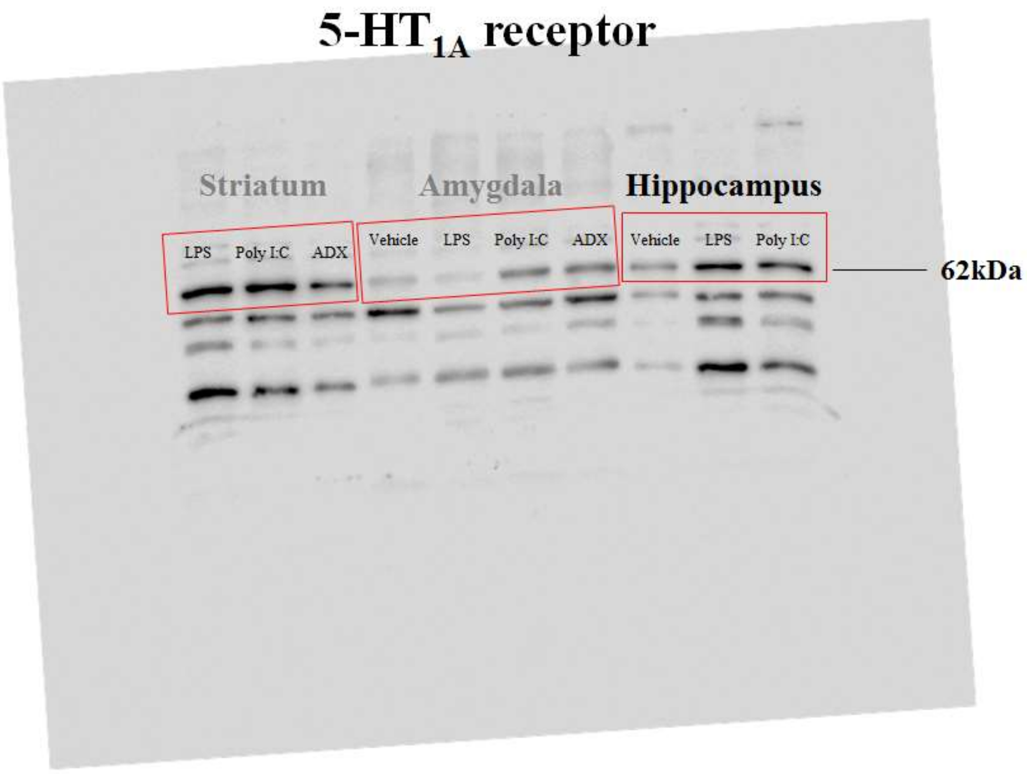

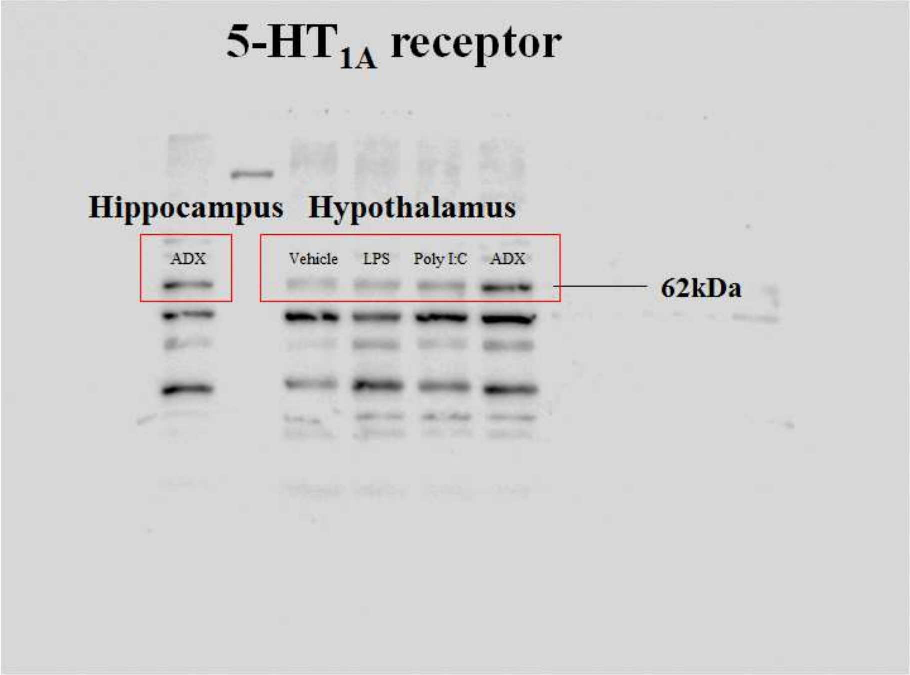
5-HT1A receptor
Hippocampus
Hypothalamus
ADX
Vehicle
LPS
Poly I:C
ADX
62kDa

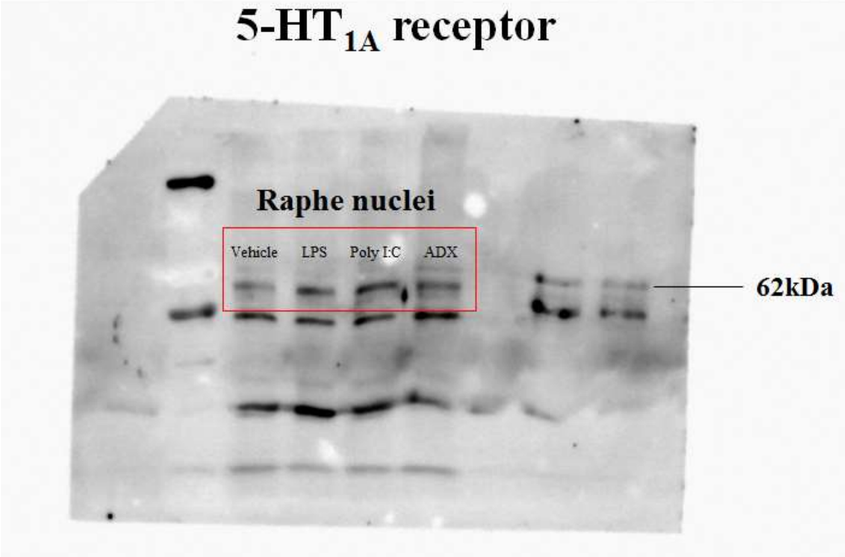
5-HT1A receptor
Raphe nuclei
Vehicle
LPS
Poly I:C
ADX
62kDa

5-HT transporter

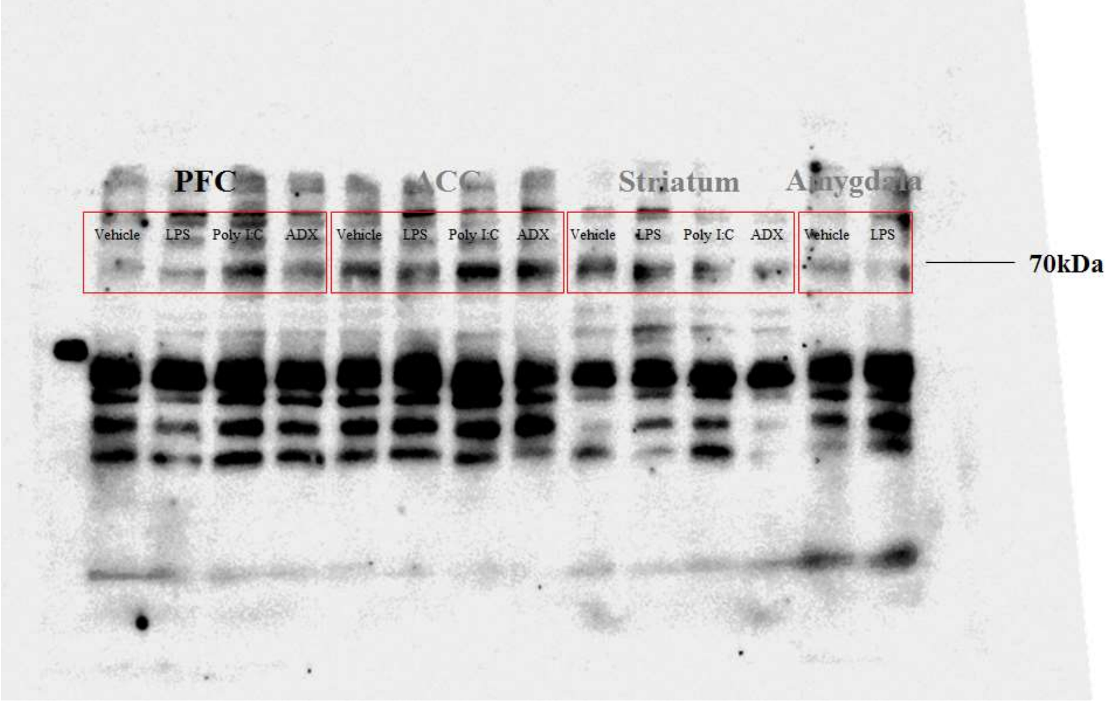

5-HT transporter

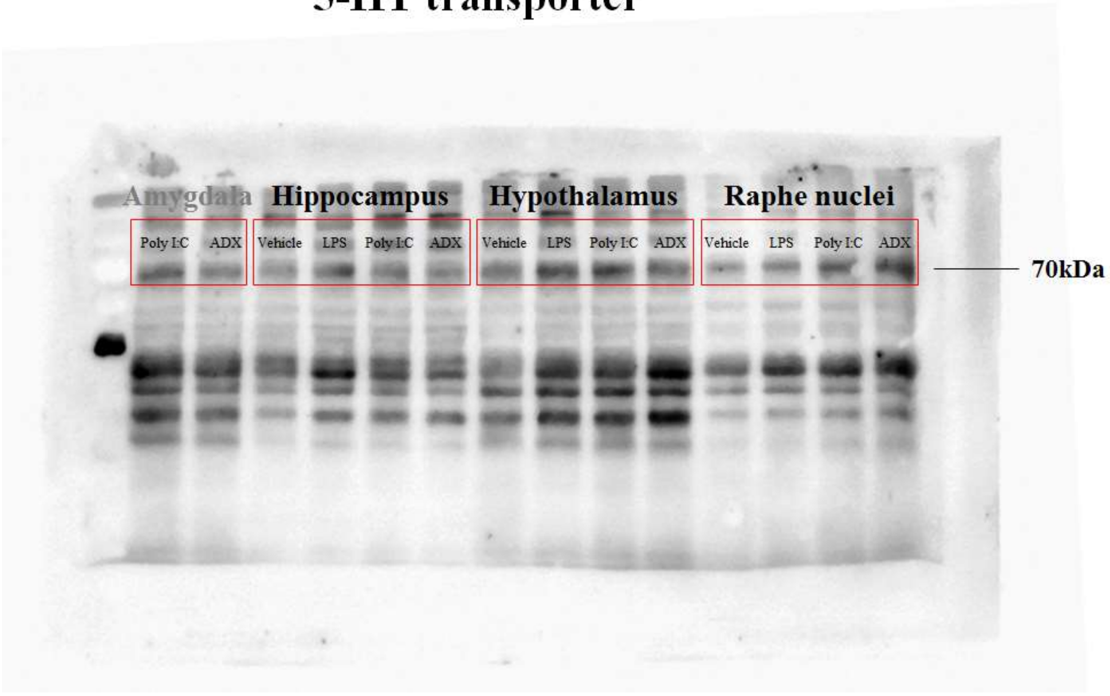

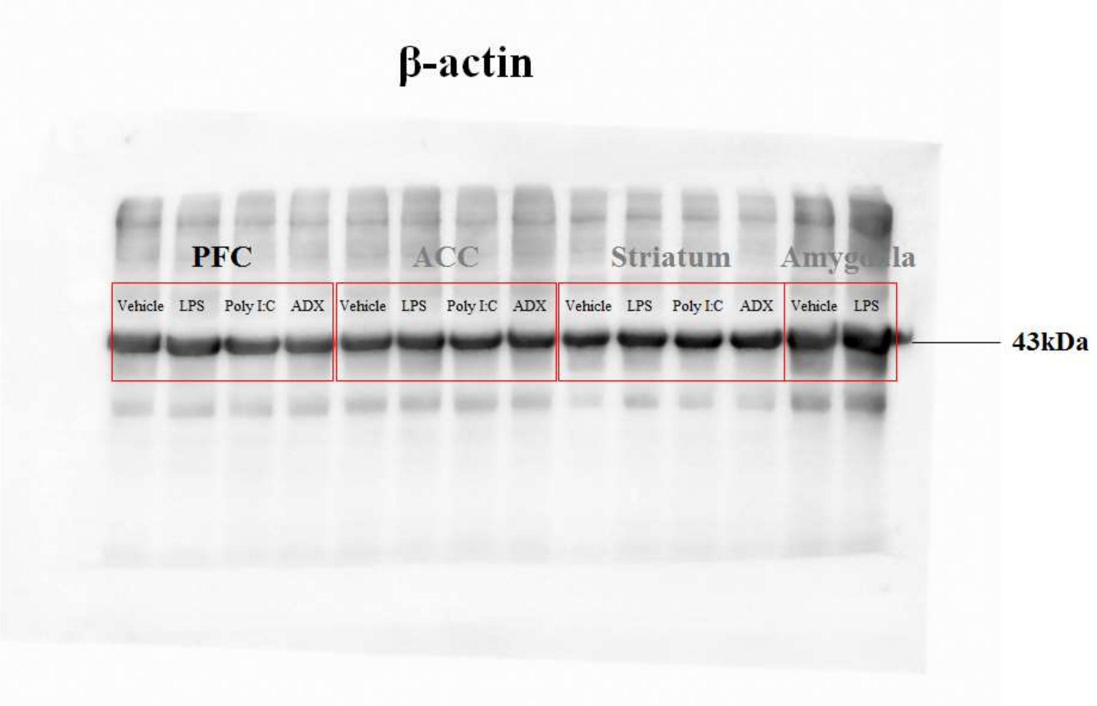
β-actin
PFC
ACC
Striatum
Vehicle LPS Poly I:C ADX Vehicle LPS Poly I:C ADX Vehicle LPS Poly I:C ADX Vehicle LPS
43kDa

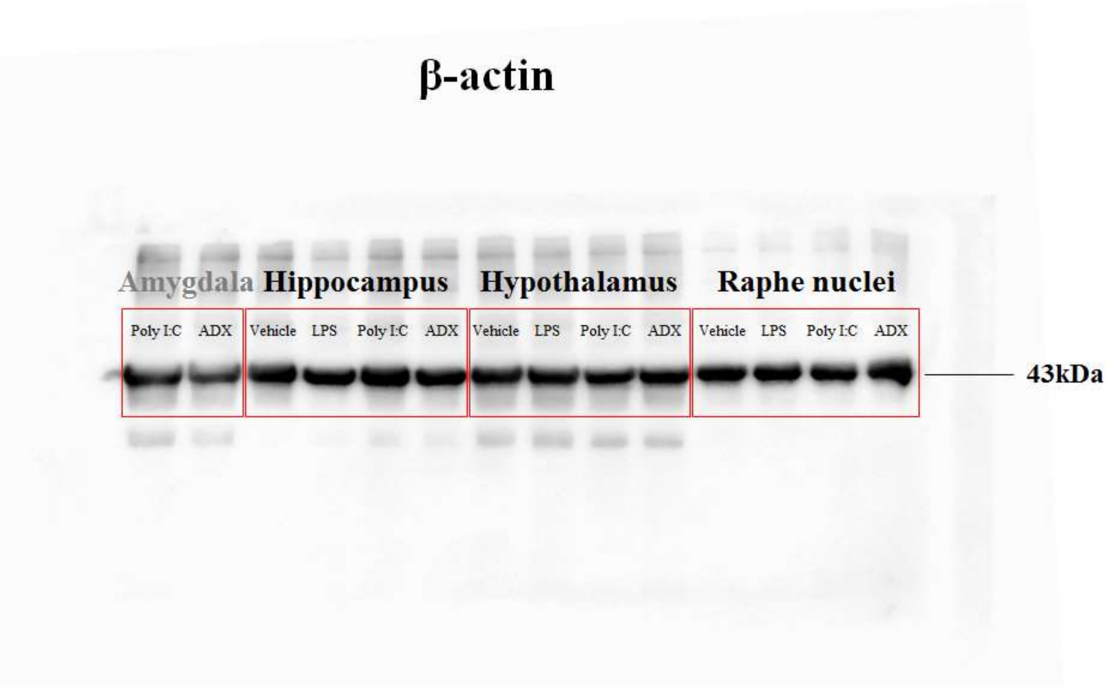
β-actin
Amygdala
Hippocampus
Hypothalamus
Raphe nuclei
Poly I:C ADX Vehicle LPS Poly I:C ADX Vehicle LPS Poly I:C ADX Vehicle LPS Poly I:C ADX
43kDa
